# The use of bioethical decision-making framework in the ICU of a public Brazilian hospital: a retrospective observational case series

**DOI:** 10.1186/s12910-026-01395-6

**Published:** 2026-03-02

**Authors:** Daniel Mendonça Dantas, Fabrício Ferreira Lipi, Sofia Carolina Cantuario de Oliveira, Matheus Parra Barcelos de Mendonça, Juliana Blassioli Suyama, Gabriel Lehvy Cantarin Gonçalves, Bernardo Ramos De Godoy, Vivian Meloni Bagini, Victor Moitinho Mariano, Ludhmila Abrahão Hajjar, Daniel Neves Forte

**Affiliations:** 1 Faculdade de Medicina, Universidade de São Paulo, São Paulo, Brazil; 2 Department of Emergency Medicine, Faculdade de Medicina, Universidade de São Paulo, São Paulo, Brazil

**Keywords:** Patient-centered care, Values-based decision, Intensive care unit, End-of-life decision-making, Withholding and withdrawal of treatment, Shared decision making

## Abstract

**Background:**

End-of-life decisions in critically ill patients lacking decisional capacity are ethically challenging. Addressing patient values—such as autonomy, dignity, and comfort—provides a person-centered basis for care, yet systematic value assessment remains inconsistent. Structured family conferences guided by a bioethical framework may help align treatment with patient preferences. This study describes how often life-sustaining treatment (LST) modifications in a Brazilian ICU were aligned with documented patient values.

**Methods:**

This retrospective observational case series reviewed electronic medical records of neurologically incapacitated adults admitted to a tertiary public ICU in São Paulo, Brazil (February 2022–August 2023). Eligible patients met predefined palliative care triggers. Data were extracted using standardized forms, focusing on demographics, clinical features, family conferences, decisions, and outcomes. Family conferences followed a structured bioethical framework and a communication model based on neurobiology to elicit patient values, which were categorized into predefined domains. Descriptive analyses were performed. The study followed STROBE guidelines, was ethics-approved with consent waiver, and did not influence clinical care.

**Results:**

Of 179 consecutive intensive care unit admissions screened, 40 patients met eligibility criteria. Most were male (65%) with a mean age of 63.5 years. Palliative care triggers included advanced age with severe comorbidities or multi-organ failure (32.5%) and cerebral ischemia (32.5%). Family conferences occurred in 57.5% of patients, typically within three days, involving sons or spouses. Patient values were documented in 50% of the cases. Of the total sample (*n* = 40), the most frequently reported values were autonomy (37.5%) and avoidance of suffering (12.5%). Overall, 55% had LST modified—withholding (50%), withdrawal (7.5%)—and 42.5% of modifications were documented value-based, representing 73.9% of patients with family conferences. Outcomes included death with LST (25%), natural death after withholding/withdrawal (25%), discharge home (25%), hospice (20%), and ward transfer (5%).

**Conclusions:**

Structured family conferences supported by a bioethical and communication frameworks were associated with a high frequency of treatment decisions explicitly aligned with patient values. These findings demonstrate that systematic value-based decision-making is feasible in resource-limited public hospitals. Prospective multicenter studies are needed to confirm these results and evaluate their impact on quality of care and clinical outcomes.

**Supplementary Information:**

The online version contains supplementary material available at 10.1186/s12910-026-01395-6.

## Introduction

End-of-life (EOL) decision-making for critically ill patients who lack decisional capacity remains a complex clinical and ethical challenge. Although tools such as advance directives are well-established in many countries, in Brazil, they are often unavailable at the time of decision-making. Increasingly, patient values have been proposed as a guide for EOL decisions, as they emphasize the individual’s priorities, fears, and trade-offs [1]. Building on this concept, the framework of “value awareness” has emerged, highlighting the importance of ensuring that decisions regarding life-sustaining treatments (LST) align with the core values of the patient rather than relying solely on clinical prognosis or surrogate interpretation [2].

However, determining whether LST decisions are truly value-based remains challenging. Even when clinicians and families strive to honor patient priorities, these values are often implicit rather than systematically addressed and documented. This gap underscores the need for structured approaches to make the process explicit, allowing both clinicians and researchers to reliably identify and quantify value-based decisions [3]. The Bioethical frameworks for decision aid in critically ill patients have been proposed to guide family conferences (FC) [4]. When applied along communication strategies, it may function both as tools for value elicitation and as mechanisms to promote high-quality, person-centered care [4, 5]. This bioethical framework has been used in family conferences at Hospital das Clínicas (HC-FMUSP), São Paulo, Brazil, since before 2018, when it was published with the onset of the COVID-19 pandemic in 2020, a structured communication strategy was introduced. This period was marked by a highly mistrustful environment and a reduced capacity for patients to participate in decision-making, which prompted the development of communication strategies aimed at better eliciting patient values through family members and rebuilding trust. Since then, these strategies have been integrated into the stepwise reasoning of the bioethical framework to provide a structured pathway toward goal-concordant care [4, 5].

Aligning LST decisions with patient values is a fundamental principle of bioethical care for critically ill patients. Nonetheless, little is known about how often LST is actually modified based on explicitly elicited values, particularly in Brazil. The present study aims to address this gap by describing the proportion of intensive care unit (ICU) patients in whom LST was modified following structured value exploration. We adopted a case series design to provide a detailed characterization of this phenomenon - value-based LST decision - in an understudied population: neurologically unable to make decisions ICU patients with palliative care triggers in Brazil. This approach allowed us to capture the clinical and ethical dimensions of these decisions while respecting the constraints inherent to EOL research.

## Methods

### Study design and setting

This retrospective observational case series reviewed electronic medical records of patients admitted to the ICU of the Emergency Department at the Central Institute of Hospital das Clínicas (HC-FMUSP), São Paulo, Brazil. The study followed the Strengthening the Reporting of Observational Studies in Epidemiology (STROBE) guidelines and was approved with a waiver of informed consent by the HC-FMUSP ethics committee (CAAE80529323.1.0000.0068, December 13, 2024). Patient data were handled confidentially, and the study did not affect clinical care.

### Population and sample

We reviewed medical records of all patients admitted to the adult ICU of the Emergency Department in a tertiary Brazilian hospital from February 2022 to August 2023. Eligible patients were neurologically unable to make independent decisions and met at least one predefined trigger for palliative care: ICU admission after > 10 days of hospitalization; age > 80 years with ≥ 2 life-threatening comorbidities or, at any age, multi-organ failure; stage IV malignancy; cerebral ischemia; family request; anticipated death during the current hospitalization; ICU stay > 1 month; estimated survival ≤ 6 months; Glasgow Coma Scale score ≤ 8 in patients > 75 years; or advanced dementia. We excluded patients in custody; without an available family member or legal proxy; admitted exclusively to surgical beds; transferred to another ICU; or with ICU length of stay < 24 h. Palliative care triggers were defined using a trigger-based model for palliative care consultation [6]. The sample included all patients meeting these criteria during the study period.

### Data source and collection

Data were extracted from the hospital’s electronic medical records (EMR) by graduate medical students supervised by senior medical students, using a standardized electronic case report form. Uncertainties were resolved through consultation with the PhD-level study supervisor. To ensure consistency and inter-rater reliability, a PhD-level senior researcher provided standardized training sessions weekly for several months before and during data collection. Extracted data included patient demographics, clinical characteristics, life-sustaining interventions, and outcomes. Family-related variables comprised demographics, relationship to the patient, and attendance at meetings. FC were documented with respect to timing from ICU admission, frequency, format, expressed patient values, decisions regarding life-sustaining therapy, consensus status, emotional concordance, healthcare professionals present, duration, and observed family dynamics.

### Statistical analysis

Descriptive statistical methods were applied using RStudio version 2024.09.1 + 394. Continuous variables were presented as mean ± standard deviation or median with interquartile range (IQR). Categorical variables were expressed as absolute numbers and percentages. A Large Language Model - ChatGPT (version 5; OpenAI) - was used only for fluency and grammar review of author-written text. No content, analyses, or conclusions were generated by the model. All authors reviewed and take full responsibility for the manuscript content.

### Patient values and preferences

**Fig. 1:**
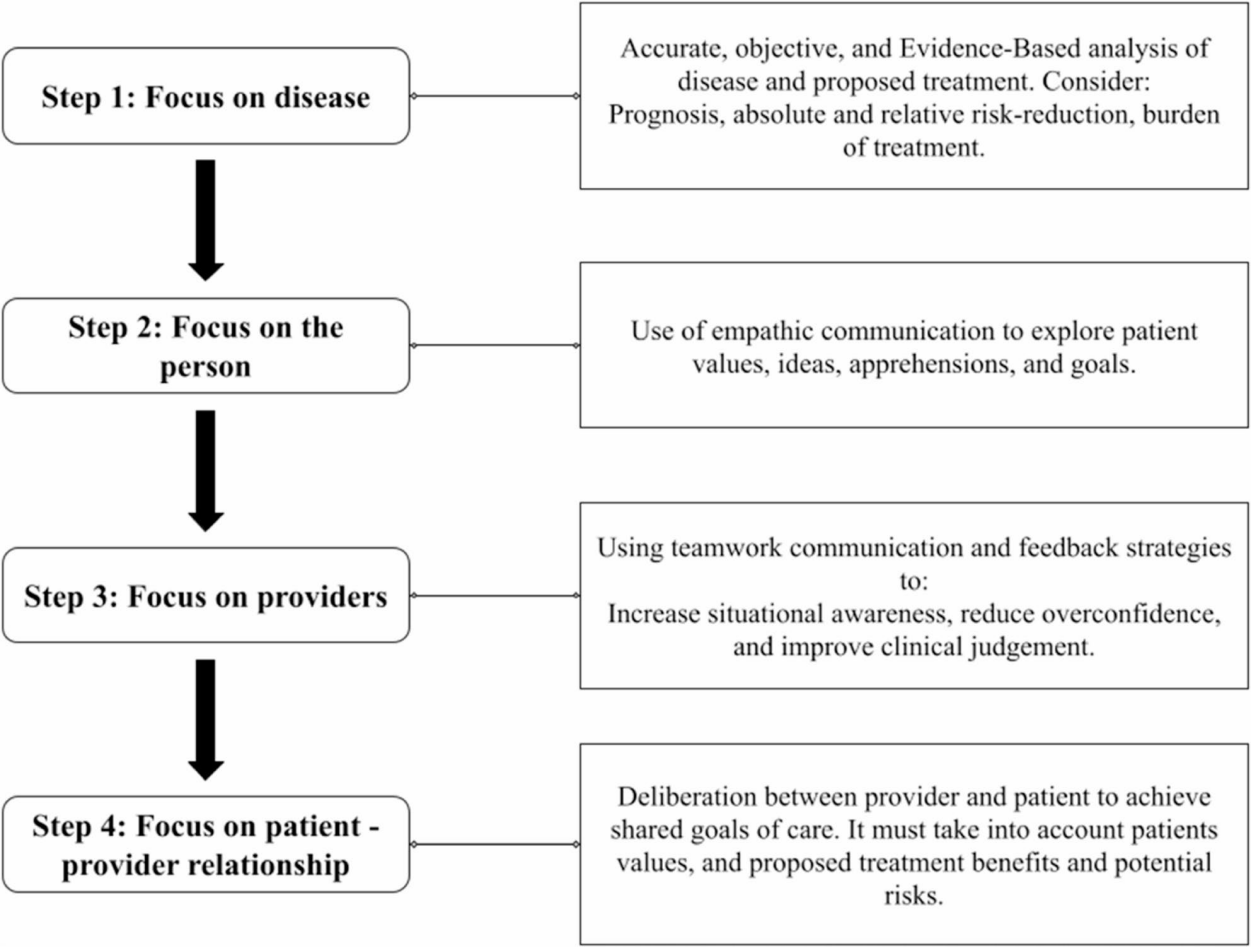
Bioethical framework for decision aid in critically ill patients from ref 4 Forte DN. et al., (2018) [4] A bioethical framework to guide the decision-making process in the care of seriously ill patients. BMC Med Ethics

**Fig. 2:**
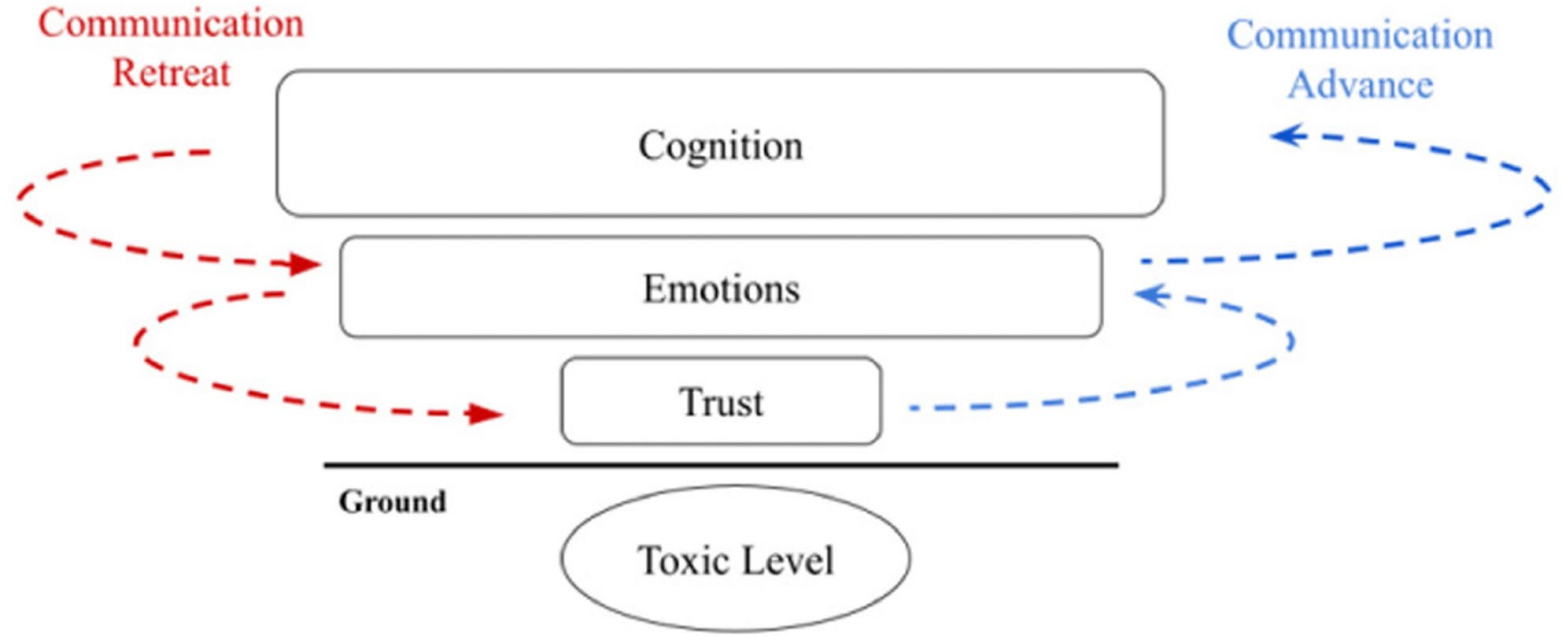
Stepwise approach to communication in family conferences and Hierarchical levels of communication and strategies to reduce mistrust from ref 5 Forte DN (2024) [5] The Hierarchy of Communication Needs: A Novel Communication Strategy for High Mistrust Settings Developed in a Brazilian COVID-ICU

Patient preferences in this study were operationalized as values previously expressed by patients to their family members or other individuals close to them who acted as surrogate decision-makers. To systematically capture these values, we utilized EMR documenting FC, which were conducted in the context of a bioethical framework designed to guide complex decision-making in seriously ill patients (Fig. 1) [4]. FCs were structured according to a hierarchized stepwise technique based on neurobiology previously described [5] (Fig. 2), designed to foster trust and promote emotional synthony before information sharing and deliberation in potentially distressing situations. This strategy comprises 3 communication levels, each one requiring a specific approach [5]. This structured method served both as a standardized interview process and as a value-elicitation tool [4, 5].

All FC were led by a consistent consulting physician, appropriately trained in bioethics and communication strategies, supported by rotating resident physicians, ensuring methodological consistency across encounters. Patient values were identified through the review of EMR, including interpretation of family conference quotations independently reviewed by two authors, extraction of values previously documented by the healthcare team, or classification as “not accessed” when neither quotations nor documented values were available.

Because some patients underwent multiple FC, all relevant records were aggregated at the individual patient level. To enhance data reliability, value-related quotes were independently reviewed and validated by two or more trained researchers through double data abstraction procedures. Discrepancies were resolved by consensus to ensure fidelity to the original clinical documentation.

Documented patient values were categorized into predefined domains, including Avoidance of Suffering, Autonomy, Dignity, Comfort, Lucidity, Hope (encompassing both expressed hopes and wishes), and Unspecified, when the decision was guided by value, but the value was not explicitly specified in the electronic report. These categories formed the basis for subsequent qualitative and quantitative analyses.

### LST decisions

Withhold decisions were defined as explicit recorded choices to not initiate or to limit advanced life-support interventions. Decisions from multiple FC were aggregated into predefined categories: Non-invasive Ventilation, Invasive Mechanical Ventilation, Renal Replacement Therapy, Vasoactive Drugs, Cardiopulmonary Resuscitation (CPR), Nasoenteral Tube, and Blood Transfusion. Absence or lack of documentation was categorized as “None.” Percentages could exceed 100% due to multiple decisions per patient.

Withdrawal decisions involved documented discontinuation of previously initiated life-sustaining interventions, based explicitly on family conference records. In this study, invasive mechanical ventilation was the only intervention discontinued. Therefore, the analyzed categories included ‘None’ and ‘Invasive Mechanical Ventilation.

### Value-based decisions

Decisions were classified as value-based when explicitly associated with documented patient values during FC. This method was adopted recognizing that the FC were based on the bioethical decision-making framework for critically ill patients described by Forte et al. (2018) [4, 5]. Thus, in order to make changes in advanced life support measures, patients’ values needed to be extracted and integrated in the proposed goals of care.

The modifications to LST were considered value-based if explicitly documented alongside patient values during FC. Decisions were classified as ‘not value-based’ when a LST decision was made without documentation of patient values or a LST decision was made in absence of a prior FC.

### Time from LST modification to outcome

The interval from the initial LST modification decision (withholding or withdrawing) to patient death was calculated from medical records, specifically for ICU deaths. Potential delays in implementing comfort-focused decisions were recognized, resulting in some patients maintaining invasive measures until death.

## Results

**Fig. 3:**
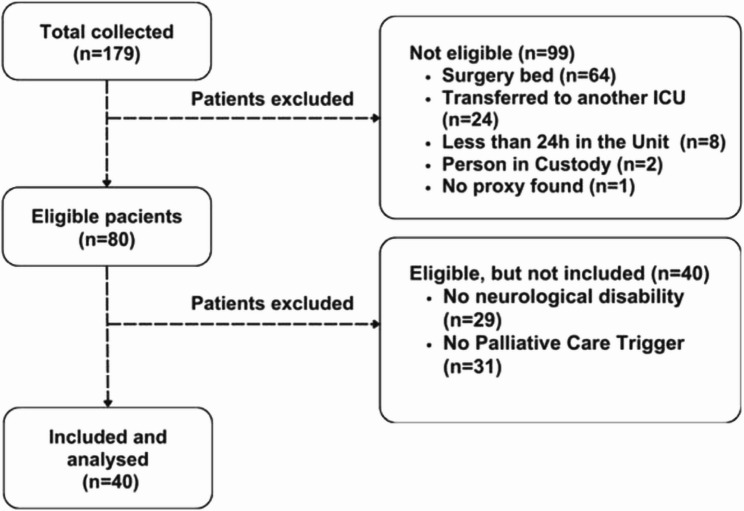
Study flowchart

During the study period, 179 ICU patients’ records were reviewed, with 40 being included in the final analysis (Fig. 3).

### Population characteristics and clinical measures before family conference

Patients’ demographic and clinical characteristics (Table 1) were as follows: the study population was predominantly male (65%) with a mean age of 63.5 years. Main palliative care triggers included advanced age with severe comorbidities or multi-organ failure (32.5%) and cerebral ischemia (32.5%), with neurologic events as the most common ICU admission reason (50%). Before FC, 70% received LST and 12.5% had LST limitations. Among the 28 patients undergoing LST before FC, 50% used invasive mechanical ventilation, 12,5% hemodialysis and 7,5% had received cardiopulmonary resuscitation.

**Table 1:** Clinical and demographic characteristics of patients (*n* = 40)

Categories	Total (*n* = 40)	Percentage
Section 1 - Gender, age at admissions, schooling, religions		
Gender		
Female	14	35,0%
Male	26	65,0%
Age at admission		
Mean, years	63,5	
Schooling		
> 13 years	3	7,5%
> 9 years	2	5,0%
9 years	12	30,0%
< 9 years	4	10,0%
Unspecified	19	47,5%
Religion		
Catholic	7	17,5%
Evangelic	1	2,5%
Other religion	16	40,0%
Unspecified	16	40,0%
Sect. 2 - Comorbidities, Previous Functionality, Palliative Care Trigger		
Previous Functionality		
Independent	17	42,5%
Partially Dependent	14	35,0%
Fully Dependent	2	5,0%
Unknown	7	17,5%
Palliative Care Trigger		
Age over 80 years with two or more life-threatening comorbidities	3	7,5%
Failure of three or more organ systems	10	25%
Cerebral ischemia	13	32,5%
Anticipated death during the current hospitalization	10	25,0%
Admission to the ICU after more than 10 days of hospitalization	7	17,5%
ICU stay exceeding one month	3	7,5%
Diagnosis with an estimated survival of six months or less	3	7,5%
Glasgow Coma Scale (GCS) score ≤ 8 for more than one week in patients over 75 years old	2	5,0%
Advanced stage of dementia	1	2,5%
Sect. 3- Reason to ICU Admission, LST limitation before ICU admission		
Reason to ICU Admission		
Neurologic	20	50,0%
Cardiovascular	16	40,0%
Renal failure	10	25,0%
Respiratory failure	10	25,0%
Sepsis	2	5,0%
Cirrhosis Decompensation	1	2,5%
Unspecified	1	2,5%
LST limitation before ICU admission	5	12,5%
Invasive Mechanical Ventilation	3	60,0%
Vasoactive drug	3	60,0%
Hemodialysis	2	40,0%
Cardiopulmonary resuscitation	3	60,0%
Sect. 4 - Use of LST before Family Conference		
Use of any LST before Family Conference	28	70%
Invasive Mechanical Ventilation	20	50%
Vasoactive drugs	23	57,5%
Previous Cardiopulmonary resuscitation	3	7,5%
Hemodialysis	5	12,5%

Table 1. Clinical and demographic characteristics of the 40 patients included in the study. Data are presented as absolute numbers and percentages, they include sex, mean age at admission, education level, religion, comorbidities, previous functionality, palliative care triggers

*ICU* Intensive Care Unit, *LST *life-sustaining treatments

### Family conferences and patient values assessment

FC characteristics, patient value, LST decisions and outcomes are presented in Table 2. FC were conducted for 57.5% of patients, involving a median of 2 family members and 3 staff; emotional resonance (Fig. 2.B) was described in 37,5% of cases. Patient values were documented in 50% of the cases. Of the total sample (*n* = 40), the most frequently reported values were autonomy (37.5%) and avoidance of suffering (12.5%). For patients undergoing family conferences, the median time to first meeting was 3 days after admission.

**Table 2:** Family conferences, patient values, LST decisions and outcomes

Category	Total (*n* = 40)	Percentage
Section 1 - Family Conferences		
Family Conference		
Yes	23	57,5%
No	17	42,5%
Number of family members		
Median (Q1, Q3) (m, M), people	2 (1, 3) (1, 14)	
Family member		
Son	16	40,0%
Spouse	8	20,0%
Siblings	9	22,5%
Parent	1	2,5%
In-laws	7	17,5%
Friend	1	2,5%
Number of hospital staff		
Median (Q1, Q2) (m, M), doctors	3 (2, 3) (1, 4)	
Emotional Resonance described in FC		
Yes	15	37,5%
No	1	2,5%
Unspecified	7	17,5%
Consensus		
Yes	20	50,0%
No	3	7,5%
Unspecified	17	42,5%
Time until consensus		
Median(Q1, Q3) (m, M), Days	3 (1, 5.5) (0, 30)	
Numbers of Family conferences		
1	12	30,0%
2	6	15,0%
3	3	7,5%
4	2	5,0%
No family conference	17	42,5%
Sect. 2 - Patient Values		
Patient values accessed		
Yes	20	50,0%
No	20	50,0%
Patient values		
Autonomy	15	37,5%
Lucidity	2	5,0%
Avoid Suffering	5	12,5%
Dignity	2	5,0%
Comfort	1	2,5%
Hope	1	2,5%
Unspecified value ^a^	2	5,0%
Sect. 3 - Clinical decisions		
Withhold of LST		
Yes	20	50,0%
No	20	50,0%
Type of LST withheld		
CPR (resuscitation)	18	45,0%
Vasoactive drugs	16	40,0%
Invasive Mechanical Ventilation	15	37,5%
Renal replacement therapy	15	37,5%
Non-invasive ventilation	2	5,0%
Nasoenteral tube	1	2,5%
Blood transfusion	1	2,5%
None	20	50,0%
Withdraw of LST		
Yes	3	7,5%
No	37	92,5%
Type of LST withdrew		
Invasive Mechanical Ventilation	3	7,5%
None	37	92,5%
Any LST modifications		
Yes	22	55,0%
No	18	45,0%
Sect. 4 - Outcomes		
Death with LST	10	25%
Natural death	10	25%
Hospice discharge	8	20%
Home discharge	10	25%
Transferred to ward	2	5%
Hospital length of stay		
Median(Q1, Q3) (m, M), Days	17.5 (10.5, 30.5) (2,140)	
Length of stay in ICU		
Median(Q1, Q3) (m, M), Days	12 (5.75, 20) (1, 121)	

Data are presented as absolute numbers and percentages. The number of family members and hospital staff was grouped per family conference. The numbers and details of family members were grouped per patient. Data on emotional syntony were analyzed only among patients who had a family conference. The total percentage of withhold and withdrawal decisions exceeds 100% because a single patient could have received more than one type of life-sustaining treatment. Number of family members, hospital staff, time to consensus, and length of stay are reported as medians with interquartile ranges (Q1–Q3), along with minimum (m) and maximum (M) values

^a^Unspecified value: decision guided by value, but value not explicitly specified in the electronic report

### LST Modifications, time metrics and clinical outcomes

LST modifications, time metrics, and clinical outcomes are summarized in Table 2. LST modifications occurred in 55%, with withholding decisions (50%) more frequent than withdrawal (7.5%). Median hospital and ICU stays were 17.5 and 12 days, respectively. ICU mortality was 50%, nearly half (42,5%) following LST modifications, with remaining patients discharged home (25%), to hospice (20%), or wards (5%).

**Fig. 4:**
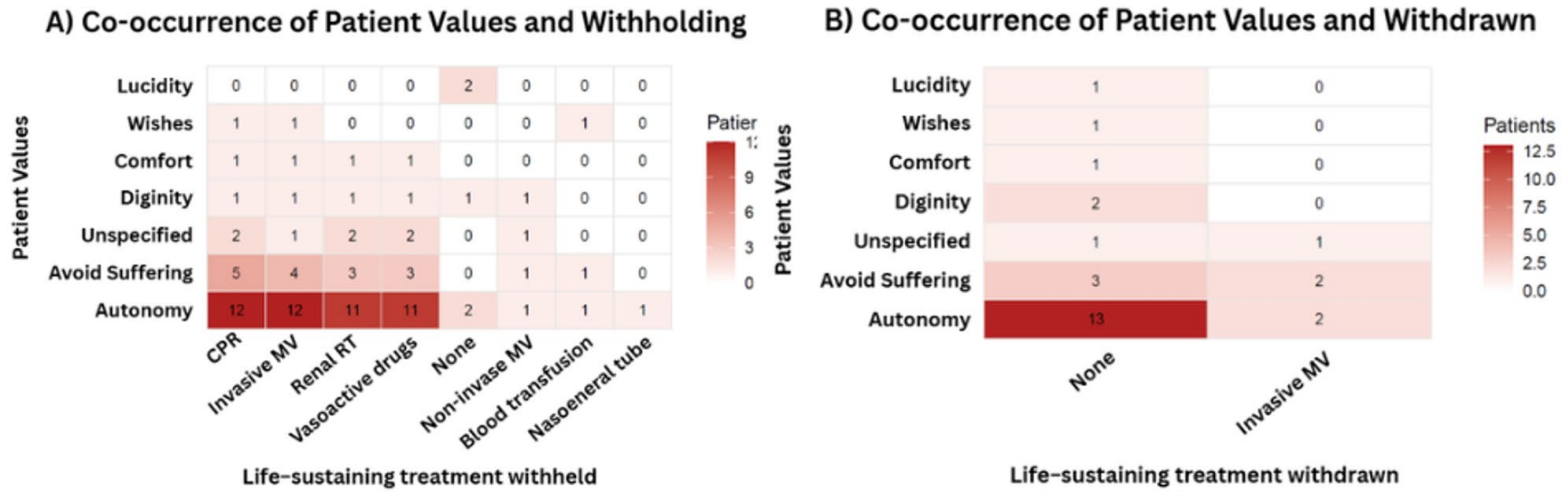
Heatmap depicting the co-occurrence of documented patient values and value-based withholding (**A**) and withdrawal (**B**) of life-sustaining treatment (LST)

Value-based clinical decisions, their outcomes and time metrics are presented in Table 3. In this study, 42.5% of LST modifications were explicitly value-based. Autonomy was the most frequently documented value and co-occurred more with withholding of LSTs, such as cardiopulmonary resuscitation, invasive mechanical ventilation, renal replacement therapy, and vasoactive drugs (Fig. 4.A). Withdrawal was uncommon and occurred exclusively in relation to invasive mechanical ventilation; in these cases, autonomy and avoidance of suffering were the most frequently documented values (Fig. 4.B).

**Table 3:** Value-based decisions and outcomes

Category	Total (*N*=40)	Percentage
Section 1 - Value-based clinical decision		
Any LST modification based on the patient's value^a^		
Yes	17	42,5%
No	23	57,5%
Section 2 - Outcome in patients with any LST modification based in patients values		
Deaths with LST	2	5%
Natural death	9	22,5%
Hospice discharge	5	12,5%
Transferred	1	2,5%
Time between LST modification based in patients values and death		
Median(Q1, Q3) (m, M), Days	6(3, 8)(1, 13)	
Time between LST modification based in patients values and death by ortotanásia		
Median(Q1, Q3) (m, M), Days	7(3, 9)(2, 13)	
Time between LST modification based in patients values and death with LST		
Median(Q1, Q3) (m, M), Days	3,5(2,25; 4,75)(1, 6)	

Data are presented as absolute numbers and percentages. Length of stay variables and time intervals between life-sustaining treatments modification and death were calculated as medians with interquartile ranges (Q1–Q3) and reported with minimum (m) and maximum (M) values. Natural death is included within overall deaths

^a^Any LST modification based on the patient’s values*: not value-based* decisions were combined with cases of no LST modification to form the ‘No’ category for the dichotomous variable *‘Any LST modification based on the patient’s values’* in Table 3, as the outcome of interest was the presence of value-based modification

## Discussion

In this retrospective study of ICU patients with palliative care triggers and unable to make their own decisions, 42.5% underwent LST changes explicitly guided by their values, elicited through structured FC—highlighting the potential of such processes to better align end-of-life care with patient preferences.

The frequency of value-based decisions observed in our series (42,5%) is higher than that reported in previous studies. Scheunemann et al. (2019) [3] described an 8.2% rate in which clinicians made treatment recommendations based on patients’ values and preferences, although in 35.7% they had important end-of-life considerations discussed and in 44.3% they deliberated about how the patients’ values applied to the decision. Our higher frequency may reflect the use of the bioethical framework in FCs structured by communications strategies, such as hierarchy of communicational needs. Unlike prior studies, we did not differentiate substituted judgment from treatment recommendations, as our FCs emphasized goal-oriented care. Notably, FCs occurred in 57.5% of patients, enabling value elicitation in half of the study population.

Furthermore, in our study, 50% of patients had life-sustaining treatments withheld and 7.5% had treatments withdrawn, findings broadly consistent with the Latin American data from ETHICUS-2 [7], which reported 61% and 6%, respectively. These regional patterns are thought to reflect well-known ethical and cultural hesitations regarding active discontinuation of therapies, as well as the limited legal frameworks governing end-of-life processes in many Latin American countries [8]. Although our study did not demonstrate substantial differences in the distribution of withholding and withdrawal compared with ETHICUS-2, it is remarkable that nearly half of the LST modifications in our series were explicitly guided by documented patient values. This alignment underscores the potential for rigorous and ethically grounded palliative care practices, even within the structural and cultural challenges characteristic of resource-limited environments.

In addition, the use of structured FC oriented by a bioethical framework allowed patient values to be translated into concrete treatment considerations, complementing more general expressions of values and preferences, consistent with previously described goal-oriented ICU communication frameworks [4, 9]. Selective withholding of high-burden life-sustaining treatments—particularly cardiopulmonary resuscitation, invasive mechanical ventilation, renal replacement therapy, and vasoactive drugs—was most commonly observed in cases where autonomy was explicitly documented, with comfort and avoidance of suffering appearing less frequently. Less invasive or supportive therapies were rarely limited. By contrast, withdrawal of ongoing treatments was uncommon and confined to invasive mechanical ventilation, a pattern that likely reflects contextual and regulatory influences on end-of-life practice in Brazil, where withdrawal decisions require explicit documentation and are subject to heightened legal scrutiny. Overall, this descriptive pattern aligns with prior evidence linking structured clinician–family communication to values-informed decision-making in critical care [10].

These findings should be interpreted within the Brazilian context, where formal advance care planning (ACP), although supported by professional regulation, remains uncommon in routine clinical practice [11, 12]. In our cohort, no patient had prior ACP documentation available at the time of ICU admission, reflecting the persistent structural, cultural, and educational barriers to end-of-life discussions in Brazil [12]. In this setting, decision-making frequently occurs in the context of acute clinical deterioration, requiring real-time clarification of patient values. The use of structured family conferences (FCs) allowed values to be elicited, documented and applied directly to LST decisions under conditions of uncertainty, providing an bioethically grounded approach to end-of-life care in the absence of prior planning. Moreover, cultural characteristics of Brazilians and many other cultures rely on the role of expressing emotions as a significant part of interpersonal encounters. Despite that, usually clinicians do not learn how to build trust and calm down patients and relatives before cognitive deliberations take place. This incongruity between culture and practice may prompt many conflicts [5]. Our data suggest that addressing emotions early in the conversation through a structured communication strategy may be one of the requirements to enable consensus in those emotionally charged situations.

The retrospective observational case-series design was appropriate given the scarcity of prior quantitative data on value-based LST modifications in neurologically impaired of making decisions ICU patients using structured tools. This approach allowed systematic description of decision-making patterns, documentation of clinical practices without intervention, and establishment of baseline data for future research, while respecting the ethical sensitivities of end-of-life care.

This study has several limitations. Its small sample size, retrospective design, and single-center setting limit generalizability, while reliance on medical records introduces the risk of incomplete or inconsistent documentation. Interpretation of patient values and reliance on consensus among clinicians may also limit reproducibility. Information bias was possible given variability in documentation by multiple physicians. To mitigate these challenges, we applied a structured supervision and training protocol, including weekly PhD-led sessions, which helped minimize inter-observer variability, and we reduced sampling bias by consecutively including all ICU admissions within a predefined period.

Despite these limitations, our findings carry important clinical implications. That nearly half of patients had care plans explicitly aligned with their values underscores the importance of structured FCs and systematic training of multidisciplinary teams in end-of-life communication, particularly in resource-limited, high-complexity settings. Future prospective studies evaluating this bioethical framework for end-of-life decision-making should include standardized documentation of how patient values were translated into specific life-sustaining treatment modification, including withholding or withdrawal, to strengthen and expand these findings. Importantly, the fact that these results were obtained in a public hospital in Brazil demonstrates that, even in middle-income, resource-constrained contexts, standards of palliative decision-making comparable to those reported in the broader literature are achievable.

## Conclusion

In critically ill patients at the end of life, structured FC supported by a bioethical framework were associated with a high frequency of treatment decisions aligned with patient values. Although prospective multicenter studies are warranted to confirm these findings and assess their impact on outcomes, our results demonstrate the feasibility of incorporating such structured approaches into ICU practice, even in resource-limited settings.

## Supplementary Information

Supplementary Material 1.

## Data Availability

The datasets used and/or analysed during the current study are available from the corresponding author on reasonable request.

## Abbreviations

EOL: End-of-life
LST: Life-sustaining treatment
FC: Family conference
ICU: Intensive care unit
IQR: Interquartile range
EMR: Electronic medical records
