# Supplementary material for: The use of bioethical decision-making framework in the ICU of a public Brazilian hospital: a retrospective observational case series
Source: BMC Med Ethics. 2026 Mar 2;27:71. doi: 10.1186/s12910-026-01395-6 (PMC13059424; doi:10.1186/s12910-026-01395-6)
Supplement: Supplementary file 1 — Supplementary Material 1. [file 12910_2026_1395_MOESM1_ESM.docx]

Supplementary Material for "The use of bioethical decision-making framework in the ICU of public Brazilian hospital: a retrospective observational case series”

# Supplementary Methods

## Study settings

Our study is a single centered observational study, which was conducted in the intensive care unit (ICU) of the Emergency Department at the Central Institute of Hospital das Clínicas, Faculty of Medicine, University of São Paulo (HC-FMUSP), São Paulo, Brazil. HC-FMUSP is the largest academic medical complex in Latin America, a public tertiary hospital system affiliated with the University of São Paulo, and a national and regional referral center for highly complex cases, advanced diagnostics, and specialized multidisciplinary care.

## Family conferences

Family conferences consisted of structured meetings involving one or more family members, the consulting physician (trained in the communication tools), and, when applicable, rotating residents and other members of the healthcare team. These conferences followed a defined communication strategy grounded in a hierarchy of communication needs, together with a bioethical decision-aid framework for critically ill patients. [1,2].

Each family conference lasted approximately 30 to 120 minutes. During these meetings, family members were actively listened to, and, after completing all communicational and ethical steps, a proposed goal of care was discussed. The conferences aimed to enhance family understanding of the patient’s condition, clarify prognosis, elicit patient values and preferences, and provide medical recommendations aligned with those values. Depending on clinical complexity and family needs, these steps could be addressed in a single meeting or across multiple conferences.

The healthcare team systematically documented their impressions, relevant excerpts from family discussions, and the patient’s values in the medical record. Whenever conflicts, subtle misalignments, or disagreements were identified, additional family conferences were scheduled to foster shared understanding, achieve consensus on goals of care, and continue the discussion from the point reached in the previous meeting. Medical recommendations and decisions regarding life-sustaining treatments were consistently guided by the principle of goal-concordant care.

## Value collection

Within the adopted bioethical framework, in the Hierarchy of communication needs model, values were collected after A) building trust; B) Calming down through emotional synthony; and C) deliberation. This deliberation process was done according the previous published communication models, specifically the Serious Illness Conversations [3].

In such models, values are collected after disclosing prognosis, explicitly asking relatives: what was important for the patient, how the patient was dealing with his previous health condition, and what was suffering for the patient [4].

Regarding the identification of values, values were collected through a qualitative review of the medical records documenting family conferences and clinical decision-making.

## Data Collection and Training Protocol

Data were collected by five trained graduate medical students, each supervised by a trained senior medical student. All team members participated in weekly training sessions led by a PhD-level senior researcher for a total of 15 months. These sessions covered the study protocol, operational definitions, and procedures for resolving ambiguous cases. During data collection, uncertainties were documented and reviewed in weekly consensus meetings with the PhD-level study supervisor to ensure uniform application of criteria.

All data were retrieved from the hospital’s electronic medical record (EMR) system. Reviewers examined the clinical notes, family conference (FC) documentation, and palliative care assessments. Information was entered into a standardized electronic database with built-in logic checks to minimize data entry errors.

## Standardized Electronic case Report Form

**Patient information**

- **Case Number**
- **Exclusion Criteria**
  - Does the patient meet exclusion criteria? (Yes/No)
  - Reason for exclusion:
- **Inclusion Criteria**
  - Neurologically incapable of making own decisions at any point during hospitalization (Yes/No)
  - Trigger for palliative care (at least 1)
  - Is the patient included? (Yes/No)

**Admission Information**

- Date of Birth (dd/mm/yyyy)
- Biological Sex
- Education Level
- Nationality
- Religion
- ICU Admission Date (dd/mm/yyyy)
- Reason for ICU Admission
- Comorbidities
- Use of life-sustaining supports prior to Family Conference
- LST limitations prior to ICU admission (Yes/No)
- Were limitations consensual? (Yes/No)
- Was a Family Conference held? (Yes/No)

**Family Conference Data**

- Time to first Family Conference (days)

**First ICU Family Conference**

- Date (dd/mm/yyyy)
- Format (In-person / Online / Not specified)
- Number of healthcare professionals involved
- Number of family/friends involved
- Relationship of family/friends involved
- Patient values expressed (Autonomy / Dignity / Suffering / Wishes)
- Consensus reached? (Yes/No)
- LST limitation or withdrawal decisions

**Second ICU Family Conference** *(repeat fields as above)*

**Third ICU Family Conference** *(repeat fields as above)*

**Fourth ICU Family Conference** *(repeat fields as above)*

**Fifth ICU Family Conference** *(repeat fields as above)*

**Additional Conference Information**

- Was emotional alignment performed? (Yes/No)
- Total number of conferences held
- Summary / Notes
- Was consensus obtained throughout the process? (Yes/No)
- Time to consensus (days)
- Time from ICU admission to consensus (days)

**Clinical and Outcome Data**

- Prior functional status
- Decision not to initiate LST (Yes/No)
- Decision to withdraw LST (Yes/No)
- Decision to limit LST (Yes/No)
- Time from decision to withdraw life-prolonging treatments until death (days)
- Total hospitalization duration (days)
- Total ICU stay (days)
- Clinical outcome (Recovered / Deceased / Other)
- Reported conflicts or ethical dilemmas with family? (Yes/No / Notes)

## Inclusion and Exclusion Criteria

**We included ICU-admitted patients who**:

1. Lacked decision-making capacity prior or during ICU stay, and
2. Met at least one predefined trigger for palliative care (see Table S1).

**Patients were excluded if they:**

Had no family or proxy found to participate in FC,

Were in police custody,

Were admitted exclusively to surgical service beds,

Were transferred to another ICU, or

Had an ICU length of stay <24 hours.

## Operational Definitions

Life-Sustaining Treatment (LST) Modification: Any documented limitation or withdrawal of interventions such as Non-invasive Ventilation, Invasive Mechanical Ventilation, Renal Replacement Therapy, Vasoactive Drugs, Cardiopulmonary Resuscitation (CPR), Nasoenteral Tube, and Blood Transfusion.

Palliative Care Triggers: Criteria adapted from national guidelines and institutional protocols (detailed in Table S1).

Family Conference: A structured meeting involving ICU physicians and patient surrogates, using the bioethical framework to guide the decision-making process (Forte et al., 2018) and The hierarchy of communication needs strategy (Forte et al., 2024) to elicit and document patient values.

# Supplementary Tables

Table S1. Detailed Criteria for Palliative Care Triggers

| **Palliative Care Trigger** | | |
| --- | --- | --- |
| Age over 80 years with two or more life-threatening comorbidities | 3 | 7,5% |
| Failure of three or more organ systems | 10 | 25% |
| Cerebral ischemia | 13 | 32,5% |
| Anticipated death during the current hospitalization | 10 | 25,0% |
| Admission to the ICU after more than 10 days of hospitalization | 7 | 17,5% |
| ICU stay exceeding one month | 3 | 7,5% |
| Diagnosis with an estimated survival of six months or less | 3 | 7,5% |
| Glasgow Coma Scale (GCS) score ≤ 8 for more than one week in patients over 75 years old. | 2 | 5,0% |
| Advanced stage of dementia | 1 | 2,5% |
| Advanced stage of cancer (metastatic) | 0 | 0% |
| Family request | 0 | 0% |

Table S2. Detailed patient-level associations between expressed values and the corresponding treatment decisions

| Patient | Value documented | Value | LST modification |
| --- | --- | --- | --- |
| 01 | Yes | Autonomy, Lucidity | None |
| 02 | Yes | Autonomy | Withheld CPR, vasoactive drug, hemodialysis  No withdraws |
| 03 | Yes | Values documented, but not specified^a^ | Withheld CPR, mechanical ventilation, vasoactive drugs  No withdraws |
| 04 | Yes | Avoid suffering | Withheld CPR |
| 05 | Yes | Autonomy | Withheld hemodialysis |
| 06 | Yes | Autonomy, Avoid suffering, Dignity | Withdraw Mechanical ventilation  Withheld CPR, vasoactive drugs, hemodialysis |
| 07 | No | Not accessed | Withdraw Mechanical ventilation |
| 08 | Yes | Comfort | Withheld CPR, mechanical ventilation, vasoactive drugs and hemodialysis |
| 09 | Yes | Autonomy | Withheld CPR, mechanical ventilation, vasoactive drugs and hemodialysis |
| 10 | Yes | Autonomy | Withheld CPR, mechanical ventilation and hemodialysis |
| 11 | Yes | Autonomy | Withheld CPR, mechanical ventilation and hemodialysis |
| 12 | Yes | Hope | None |
| 13 | Yes | Autonomy | Withheld CPR, mechanical ventilation, vasoactive drugs and hemodialysis |
| 14 | Yes | Autonomy | Withheld CPR, mechanical ventilation, vasoactive drugs and hemodialysis |
| 15 | Yes | Documented but not specified^a^ | Withheld CPR, vasoactive drugs and hemodialysis |
| 16 | Yes | Autonomy | Withheld CPR, mechanical ventilation, vasoactive drugs and hemodialysis |
| 17 | Yes | Autonomy and avoid suffering | Withdraw mechanical ventilation  Withheld CPR, vasoactive drugs, hemodyalisis |
| 18 | No | Not accessed | None |
| 19 | Yes | Autonomy | Withheld CPR, mechanical ventilation, vasoactive drugs and hemodialysis |
| 20 | Yes | Autonomy | Withheld CPR, mechanical ventilation |
| 21 | Yes | Autonomy, avoid suffering | Withheld CPR, mechanical ventilation, vasoactive drugs and hemodialysis |
| 22 | No | Not accessed | None |
| 23 | Yes | Autonomy, dignity, avoid suffering, Lucidity | None |
| **Table Caption**  ^a^Value documented but not specified: decision guided by value, but value not explicitly specified in the electronic report. | | | |

# Supplementary Figures

Figure S1. Flow Diagram of Patient Selection


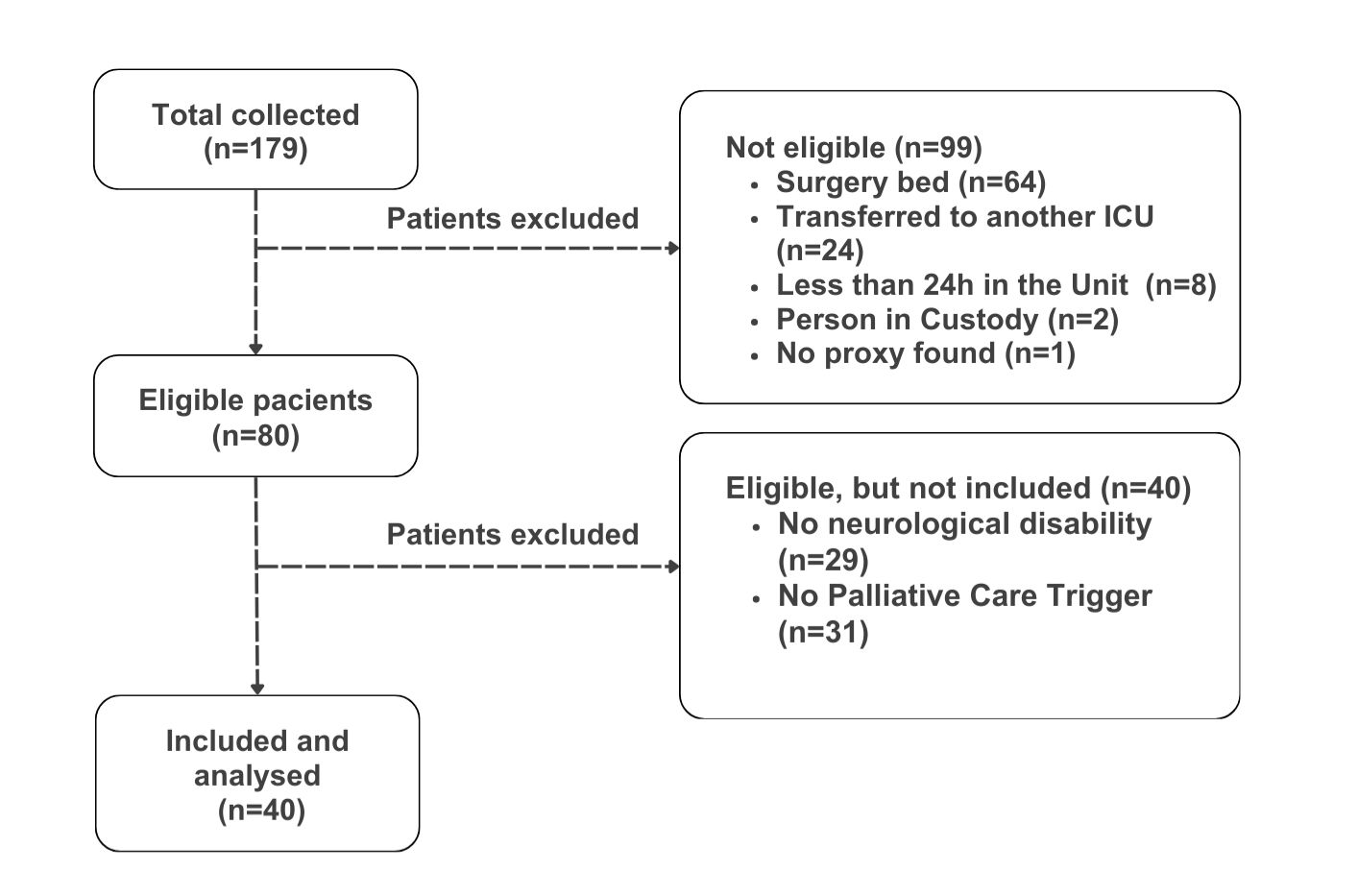


# Supplementary Statistical Analysis

**Large Language Model Assistance**

Fluency, coherence, and grammar checks were performed using ChatGPT (version 5; OpenAI). No content, analyses, or conclusions were generated by the model.

**Statistical Software**

Analyses were performed in R (version 4.4.1) using the following packages: tidyverse, ggplot2, stringr, purrr, tibble, tableone. Code for the primary analysis is available on reasonable request.

**Handling of Missing Data**

Missing values were treated as negative responses for the corresponding category. All available data were included in the analyses.

# Additional References

1. Forte DN, Kawai F, Cohen C. A bioethical framework to guide the decision-making process in the care of seriously ill patients. BMC Med Ethics. 2018;19(1):31. doi:10.1186/s12910-018-0317-y
2. Forte DN, Stoltenberg M, Ribeiro SCdC, de Almeida IM(M)O, Jackson V, Daubman B-R. The hierarchy of communication needs: a novel communication strategy for high mistrust settings developed in a Brazilian COVID-ICU. Palliat Med Rep. 2024;5(1):86–93. doi:10.1089/pmr.2023.0070
3. Daubman BR, Bernacki R, Stoltenberg M, Wilson E, Jacobsen J. Best Practices for Teaching Clinicians to Use a Serious Illness Conversation Guide. Palliat Med Rep. 2020 Jul 28;1(1):135-142. doi: 10.1089/pmr.2020.0066. PMID: 34223467; PMCID: PMC8241361
4. Scheunemann LP, Arnold RM, White DB. The facilitated values history: helping surrogates make authentic decisions for incapacitated patients with advanced illness. Am J Respir Crit Care Med. 2012 Sep 15;186(6):480-6. doi: 10.1164/rccm.201204-0710CP. Epub 2012 Jul 19. PMID: 22822020; PMCID: PMC3480534.
